# Non-Communicable Disease Preventive Screening by HIV Care Model

**DOI:** 10.1371/journal.pone.0169246

**Published:** 2017-01-06

**Authors:** Corinne M. Rhodes, Yuchiao Chang, Susan Regan, Virginia A. Triant

**Affiliations:** 1 Division of General Internal Medicine, University of Pennsylvania School of Medicine, Philadelphia, Pennsylvania, United States of America; 2 Division of General Internal Medicine, Massachusetts General Hospital, Boston, Massachusetts, United States of America; 3 Harvard Medical School, Boston, Massachusetts, United States of America; 4 Division of Infectious Diseases, Massachusetts General Hospital, Boston, Massachusetts, United States of America; University of Washington Department of Global Health, UNITED STATES

## Abstract

**Importance:**

The Human Immunodeficiency Virus (HIV) epidemic has evolved, with an increasing non-communicable disease (NCD) burden emerging and need for long-term management, yet there are limited data to help delineate the optimal care model to screen for NCDs for this patient population.

**Objective:**

The primary aim was to compare rates of NCD preventive screening in persons living with HIV/AIDS (PLWHA) by type of HIV care model, focusing on metabolic/cardiovascular disease (CVD) and cancer screening. We hypothesized that primary care models that included generalists would have higher preventive screening rates.

**Design:**

Prospective observational cohort study.

**Setting:**

Partners HealthCare System (PHS) encompassing Brigham & Women’s Hospital, Massachusetts General Hospital, and affiliated community health centers.

**Participants:**

PLWHA age >18 engaged in active primary care at PHS.

**Exposure:**

HIV care model categorized as infectious disease (ID) providers only, generalist providers only, or ID plus generalist providers.

**Main Outcome(s) and Measures(s):**

Odds of screening for metabolic/CVD outcomes including hypertension (HTN), obesity, hyperlipidemia (HL), and diabetes (DM) and cancer including colorectal cancer (CRC), cervical cancer, and breast cancer.

**Results:**

In a cohort of 1565 PLWHA, distribution by HIV care model was 875 ID (56%), 90 generalists (6%), and 600 ID plus generalists (38%). Patients in the generalist group had lower odds of viral suppression but similar CD4 counts and ART exposure as compared with ID and ID plus generalist groups. In analyses adjusting for sociodemographic and clinical covariates and clustering within provider, there were no significant differences in metabolic/CVD or cancer screening rates among the three HIV care models.

**Conclusions:**

There were no notable differences in metabolic/CVD or cancer screening rates by HIV care model after adjusting for sociodemographic and clinical factors. These findings suggest that HIV patients receive similar preventive health care for NCDs independent of HIV care model.

## Introduction

The human immunodeficiency virus (HIV) epidemic has transformed over the last 30 years, with affected individuals now achieving near-normal life expectancy[1–3]. As a result, persons living with HIV/AIDS (PLWHA) are aging, with 24% (288,700) of the estimated 1.2 million older than 55 years in 2012[4].

PLWHA have an increased burden of non-communicable diseases (NCDs) relative to HIV-uninfected patients[5, 6] with models predicting that more than a quarter of PLWHA will have three or more NCDs by 2030[6]. PLWHA also have higher incidence of specific NCDs, including non-AIDS defining malignancies[7, 8], and cardiovascular disease[9–14], and have different indications for screening for some NCDs[15]. The changing nature of comorbidities in HIV has necessitated development of HIV care models that address chronic disease management[16, 17], yet there are limited data to help determine the optimal care model for this patient population[18].

HIV care models vary by population density and geography[19–23] based on factors including specialist availability, individual provider preference, local practice patterns, and training experience. Models of HIV care include infectious disease (ID) provider acting as both HIV provider and primary care provider (PCP), generalist provider acting as both HIV provider and PCP, and/or ID provider acting as HIV provider with generalist acting as PCP.

There is limited literature on screening for or managing NCD outcomes by different HIV care models[24–26]. Compared with HIV-uninfected patients, PLWHA may be screened and treated for NCDs at lower rates[10, 13, 27, 28]. ID providers report less comfort managing diabetes (DM), hypertension (HTN), hyperlipidemia (HL) and depression than generalists[29] and have four times increased odds of referring for HTN or DM management[30]. A study examining Canadian HIV care models demonstrated HIV care models with family physicians had higher odds of cancer screening than those with exclusive specialty care[26]. In the US, however, studies comparing HIV care models with integrated ID plus generalist care to either generalist or ID care showed no differences in breast or colorectal cancer (CRC) screening rates[24, 25]. A review examining generalist and specialist care called for additional research in this sphere to help inform health policy and the optimal roles for generalist and specialty care providers[31].

Our primary aim was to compare rates of NCD preventive screening in PLWHA by type of HIV care model, focusing on metabolic/cardiovascular disease (CVD) and cancer screening. We hypothesized that HIV care models including generalists would have higher preventive screening rates concordant with guidelines.

## Materials and Methods

### Cohort Development

We used the Partners HIV cohort, a longitudinal clinical care cohort with data derived from the Research Patient Data Registry (RPDR), a Partners HealthCare System (PHS) data registry with over 5 million patients[32]. For the current study, inclusion criteria were (1) age ≥18 on 1/1/2012, (2) HIV/AIDS as defined by ≥3 ICD-9 codes for HIV (042 and all subtypes and V08) within a 5 year time frame from 1/1/2008 to 12/31/2012, and (3) active primary care in PHS defined as ≥1 visit to PCP or ID during 2012. This HIV definition was assessed by chart review in a randomly selected sample of 100 patients in the Partners HIV cohort and 100% were found to be HIV-infected.

A subgroup analysis limited to patients with at least 2 visits in 2012 ≥90 days apart, the Institution of Medicine (IOM) definition for retention in care,[33] was conducted to examine potential outcome differences between active primary care and IOM retention in care definitions. Exclusion criteria included known death in 2012 or a primary care or HIV provider outside PHS in 2011–2012. IRB approval was obtained from the Partners Human Research Committee without written informed consent. Patient records/information were not anonymized and de-identified prior to analysis.

### Exposure Classification

Detailed exposure classification with validation of HIV care models is available in Methods A and Table A in S1 Appendix. Briefly, the primary exposure of interest was type of HIV care model based on clinic visits to the corresponding type of provider(s) between 1/1/2011 and 12/31/2012: (1) infectious disease provider only (ID), (2) generalist provider only (generalist), or (3) infectious diseases and generalist providers (ID plus generalist). HIV care models underwent iterative targeted chart review and multiple random chart review validations. Final cohort HIV care model validation by random 100 person chart review identified correct exposure classification in 94% of patients. We chose not to collapse the smaller generalist group into ID or ID plus generalist as this group was thought to represent a unique care model.

### Covariates

Covariates included patient demographics, language (English vs. non-English), insurance status, recent and nadir CD4 cell counts, recent viral load, and ever use of antiretroviral therapy (ART). We calculated weighted Charlson scores[34, 35]. We estimated household income using zip code-level medians from the American Community Survey[36], and transformed median household income to percent of the state-wide median household income[37] with pre-specified cut points[38] as a socioeconomic status (SES) indicator.

### Outcomes

Our primary outcome was completion of selected NCD preventive screenings in 2012 based on the Infectious Disease Society of America (IDSA) 2009 primary care guidelines for persons infected with HIV[15]. We included annual hypertension (HTN) screen, expected to have been available for all patients, as a reference. Obesity screening was determined based on recorded body mass index (BMI) or weight in 2012 with available patient height. Patients with missing HTN or BMI data from structured vital sign fields in EMR underwent physician medical record review. Hyperlipidemia (HL) screening was ascertained using structured health maintenance fields and laboratory data, excluding non-fasting results. Diabetes screening in non-diabetic patients was ascertained by fasting glucose and A1C laboratory data, as recommended by the United States Preventative Force Task Force (USPSTF)[39]. We also assessed diabetes screening by A1C or fasting glucose testing individually. Glucose tests were considered fasting if drawn at the same time as an LDL and triglycerides were <400, adapted from a proxy algorithm[40].

For cancer screening outcomes, we adapted Massachusetts General Hospital’s Primary Care Operations Improvement (PCOI) algorithms which have been previously validated and published[41]. Patients’ eligibility and completion of breast, cervical, and colorectal cancer were in accordance with the published algorithms; however, the age range for women eligible for breast and cervical cancer screening were modified to 50–74 and 18–74 and the interval for cervical cancer modified to annual screening based on HIV primary care guidelines. Table B in S1 Appendix indicates the respective patient characteristics, exclusion criteria, and screening test criteria applied for colorectal cancer (CRC), cervical cancer, and breast cancer screening[15]. For non-annual screening tests, we examined the appropriate interval for completed tests indicated in Table B in S1 Appendix.

### Analysis

For patient demographic and clinical characteristics, we used ANOVA and Kruskal-Wallis tests to compare normally and non-normally distributed continuous variables and Chi-square tests or Fisher’s exact tests for categorical variables among our three exposure groups. To account for the correlation among patients seen by the same provider, we analyzed each screening outcome by both simple and multivariable logistic regression models using the generalized estimating equations (GEE) techniques. We used the most common provider, defined as the ID or generalist physician with the most 2012 patient encounters as the cluster in these models.

For the multivariable analysis, we included the following pre-specified covariates in the models selected on the basis of clinical importance: age, sex, race, English speaking, number of 2012 clinic visits, median household income, viral load, CD4 cell count, and weighted Charlson score. For breast cancer, we used a smaller pre-specified model due to restricted number of qualifying patients: age, English language, number of 2012 clinic visits, viral load, CD4 cell count, and weighted Charlson score. We also performed a sensitivity analysis adjusting for number of 2012 visits. We used SAS version 9.4 (SAS Institute Inc., Cary, NC). A two-sided p≤0.05 was considered statistically significant.

## Results

**Fig 1:**
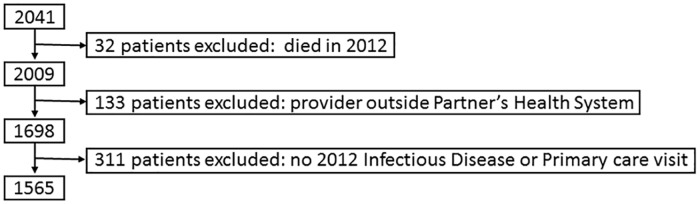
Cohort Development. The flowchart indicates specific exclusion criteria applied in a stepwise manner to develop the final cohort for the study.

From an initial cohort of 2041 PLWHA adults with ID or general medicine encounters in 2012 we excluded 476: 32 who died in 2012, 133 due to absence of ID or generalist visit in 2012, and 311 who had a designated outside provider, yielding a final cohort of 1565 patients (Fig 1).

Patients were categorized into the ID only group (n = 875), the generalist only group (n = 90), and the ID plus generalist group (n = 600), with 74 ID providers from 7 ID clinics and 311 generalists from 39 primary care clinics. Table 1 displays demographic and clinical summary statistics in each HIV care group. The ID group was 56.0% white (vs. 34.4% generalist and 42.3% ID plus generalist), 77.4% male (vs. 63% in both generalist and ID plus generalist), and had IOM retention criteria rates of 88.3% (vs. 94.4% generalist and 96.3% ID plus generalist). The generalist group’s proportion of Hispanic patients was high at 40.0% (vs. 13.8% ID and 16.7% ID plus generalist) and its proportion of English speaking patients was low at 60.0% (vs. 93.0% ID and 86.5% ID plus generalist). The ID plus generalist group had high proportions of black patients (36.0% vs. 25.6% ID and 24.4% generalist), more median visits in 2012 (6 vs. 4 in ID and 5 generalist) and higher median weighted Charlson score (9 vs. 8 in ID and 7 in generalists).

**Table 1:** Cohort Demographic and Clinical Characteristics.

Total: n = 1565*	ID n = 875	Generalist n = 90	ID plus Generalist n = 600	P value**
Age: Mean (SD)	49.9 (9.8)	47.7 (11.6)	50.4 (10.1)	0.06
Male	677 (77.4%)	57 (63.3%)	378 (63.0%)	<0.0001
Race:				
White Black Hispanic Other	490 (56.0%) 224 (25.6%) 121 (13.8%) 40 (4.6%)	31 (34.4%) 22 (24.4%) 36 (40.0%) 1 (1.1%)	254 (42.3%) 216 (36.0%) 100 (16.7%) 30 (5.0%)	<0.0001
English Speaking	814 (93.0%)	54 (60.0%)	519 (86.5%)	<0.0001
Insurance:				
Private Medicare/Medicaid Self Pay Missing	629 (71.9%) 100 (11.4%) 7 (0.8%) 139 (15.9%)	71 (78.9%) 4 (4.4%) 1 (1.1%) 14 (15.6%)	407 (67.8%) 90 (15.0%) 4 (0.7%) 99 (16.5%)	0.10
Estimated Median Household Income as Percent of Statewide median:				
<60% 60–100% 100–140% >140%	165 (19.4%) 351 (41.2%) 258 (30.3%) 78 (9.2%)	11 (12.4%) 58 (65.2%) 9 (10.1%) 11 (12.4%)	130 (22.0%) 241 (40.7%) 172 (29.1%) 49 (8.3%)	<0.0001
Median 2012 Visits (IQR)	4 (3–6)	5 (3–6)	6 (4–9)	<0.0001
IOM Retention Criteria(33)	773 (88.3%)	85 (94.4%)	578 (96.3%)	<0.0001
Recent CD4: Median (IQR)***	583 (384–795)	561 (349–840)	576.5 (390–802)	0.80
Nadir CD4: Median (IQR)***	190 (60–327)	233 (64–322)	213 (63–342)	0.49
Recent viral load <400 copies/uL***	726 (89.3%)	65 (79.3%)	484 (87.7%)	0.03
ART ever use	853 (97.5%)	87 (96.7%)	575 (95.8%)	0.22
Weighted Charlson: Median (IQR)	8 (7–10)	7 (6–10)	9 (7–11)	<0.0001

* For median household income: n = 1533; CD4 cell count: n = 1452; viral load: n = 1447, otherwise all data reflect N (%)

**P values testing overall differences using ANOVA, Kruskal-Wallis, Fisher’s exact test, or Chi squared test

***Missing laboratory data was 6.6%, 5.6%, and 6.3% respectively for ID, generalist, and ID plus generalist groups

Nadir and recent CD4 cell counts as well as ART prescription rates were similar across the three groups. The generalist group patients, however, had lower viral suppression rates (HIV viral load <400copies/uL) than ID and ID plus generalist groups (79.3% vs. 89.3% and 87.7%) with a significant Kruskal Wallis test for 3 way comparison (p = 0.03) and pairwise testing (ID vs. generalist p = 0.01, generalist vs. ID plus generalist p = 0.05).

Table 2 shows screening rates for metabolic/CVD and cancer outcomes by HIV care model. As anticipated, rates of HTN screening were high, exceeding 99% in all three groups. Other metabolic and cancer screening outcomes show variation in rates, with screening rates as low as 40% for diabetes screening, and differences in screening rates ranging from 4% (diabetic screening) to 25% (hyperlipidemia screening) when comparing HIV care models. A post hoc statistical power analysis showed >80% power to detect the following differences in each screening outcomes: ≥5% in HTN, obesity, and HL (n = 1565), ≥15% DM (n = 1222), ≥20% in CRC, ≥25% in cervical cancer, and ≥33% in breast cancer.

**Table 2:** Metabolic/CVD and Cancer Screening Rates by HIV Care Model.

	n	ID	Generalist	ID plus Generalist
Hypertension	1565	872 (99.7%)	90 (100%)	599 (99.8%)
Obesity	1565	767 (87.7%)	78 (86.7%)	556 (92.7%)
Hyperlipidemia	1565	491 (56.1%)	73 (81.1%)	366 (61.0%)
Diabetes	1222	322 (44.5%)	31 (43.7%)	172 (40.2%)
Colorectal cancer	814	275 (61.0%)	21 (53.9%)	204 (63.0%)
Cervical cancer	413	91 (47.6%)	15 (57.7%)	93 (47.5%)
Breast cancer	192	47 (61.8%)	10 (71.4%)	74 (72.6%)

**Fig 2:**
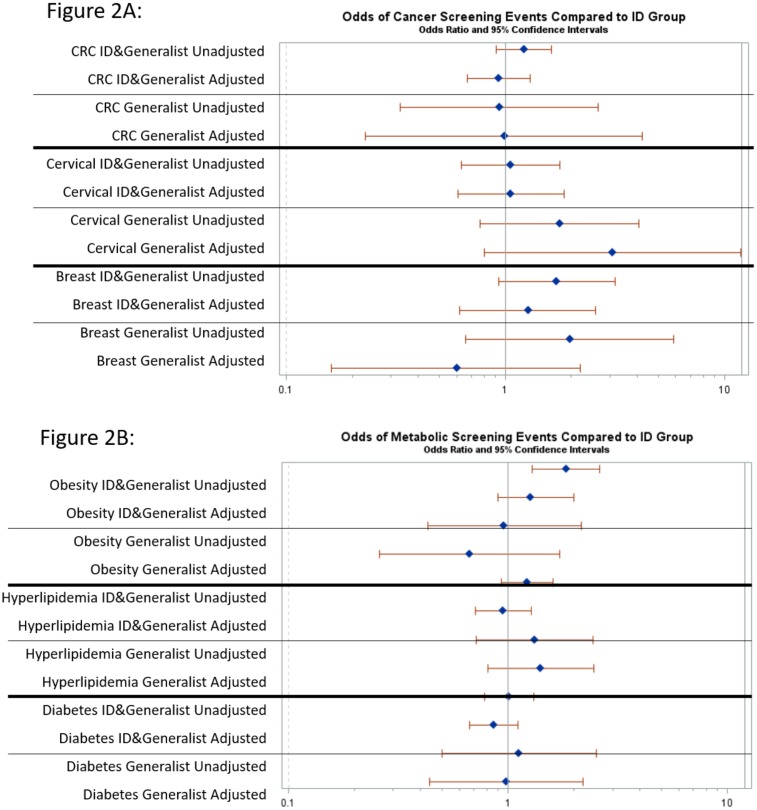
Odds of non-communicable disease screening events comparing ID plus generalist and generalist groups to ID group. Panel A shows odds of metabolic/CVD screening. Panel B shows odds of cancer screening rates. Odds ratios and 95% confidence intervals are shown for unadjusted and adjusted analyses.

Fig 2 shows odds ratios (OR) and 95% confidence intervals (CI) for each outcome using ID only as the reference group. For metabolic/CVD outcomes (Fig 2a) ID plus generalist had increased odds of screening for obesity compared with ID (OR 1.84, 95% CI 1.29–2.61), but this result was attenuated and no longer significant in the adjusted analysis. For HL and DM, there were no significant differences in screening rates between HIV care models in unadjusted or adjusted analyses. For cancer screening (Fig 2b), Colorectal, breast and cervical cancer screening rates were not significantly different between groups. Table C in S1 Appendix shows the odds ratios and 95% CI for each comparison.

The subgroup analysis including only patients who met IOM retention in care criteria (n = 1436) demonstrated similar results to the primary analyses. Sensitivity analysis removing number of 2012 visits from the adjusted model also demonstrated similar results, although ID plus generalist had significantly higher odds of screening for obesity compared to ID in both unadjusted and adjusted models (aOR 1.67 95% CI 1.1–2.54).

**Fig 3:**
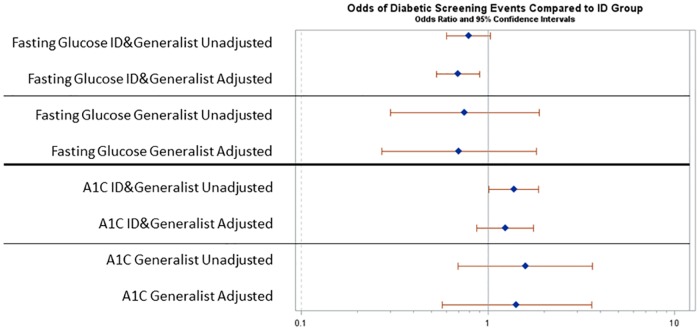
Odds of comparative diabetes screening events by specific screening types (A1C vs. fasting glucose). Odds ratios and 95% confidence intervals are shown for for unadjusted and adjusted analyses.

DM screening was further assessed by use of A1C or fasting glucose testing in an exploratory analysis of these non-mutually exclusive tests. Unadjusted rates of screening by fasting glucose were 37.6% in ID, 35.2% in generalists, and 25.2% in ID plus generalists. In adjusted analysis, odds of DM screening by fasting glucose were significantly lower in ID plus generalist versus ID (OR 0.69, 95% CI 0.53–0.90) (Fig 3). The ID group had an A1C screening rate of 12.7% compared to ID plus generalists and generalists rates of 19.7% and 18.5% respectively. In adjusted analysis, there was not a significant difference in the odds of DM screening by A1C comparing the ID with the ID plus generalist or generalist groups.

## Discussion

In a contemporary HIV clinical care cohort, odds of screening for metabolic/CVD and cancer outcomes did not differ significantly based on HIV care model, comparing patients cared for by ID providers with those cared for by generalists or by ID plus generalist. Our findings suggest that the three models of care delivery are likely to be equally effective in assuring that PLWHA receive appropriate screening for chronic disease outcomes.

Studies from the 1990s assessed HIV-related outcomes by HIV care model and showed that generalists provided equivalent care compared to ID providers. Increased experience caring for PLWHA or HIV patient volume has been shown to affect the rate of incorporating new literature into practice[42–46], HIV outcomes[47, 48], and mortality[49, 50]. Yet relatively few studies have assessed the optimal HIV care model—whether comprised of ID, generalist, or both—in terms of NCD screening in the current era of HIV care[24–26], when prevention and management of chronic disease complications are key clinical priorities.

We found no significant differences by HIV care model in metabolic/CVD and cancer screening rates, the primary outcomes of the study. These findings suggest that PLWHA are receiving similar levels of screening for NCDs regardless of their HIV care model, contrary to our hypothesis. Our data may reflect increased focus of ID care at the provider, clinic, and system level on the heightened risk of NCDs among aging HIV-infected individuals and shifting priorities of HIV care.

Our results reflect those of a study showing no difference in breast cancer or CRC screening rates by integrated clinic models in Philadelphia. In contrast, a recent study from Ontario showed HIV care models involving only specialty providers to have lower rates of cancer screening, however, it is notable that the Canadian system considers internists (the majority of our study’s generalist population) specialty providers and reported comparisons of ID and internist providers to family practitioners. Another recent study showed similar rates of cholesterol screening when compared with those from our study, among generalists (80% vs. 81.1% in our cohort) and ID specialists (54% vs. 56.1% in our cohort) but higher screening rates among ID plus generalist (77% vs. 61.0% in our cohort). We expanded on these findings by assessing multiple additional screening outcomes and applying clustering, a methodology that accounts for correlation in outcomes across patients treated by a given physician.

While screening rates for HTN and obesity were high, screening rates for other NCDs were lower and not guideline-conforming[15]. However, comparing cancer screening rates to 2010 national screening rates[51], HIV care models were similar or above national screening rates for CRC (58.6%), breast cancer (72.4%) (Table 2). National cervical cancer screening rates in the prior 3 years are 83%[51] but reflect a recommendation to screen every three years; in contrast cervical cancer screening rates ranging from 47–58% occurred in the context of a recommendation for annual screening[15] in PLWHA.

HIV care models have varied over time and by clinical setting. In our cohort, the most common HIV care model was ID (56%) followed by ID plus generalist (38%), with a minority of patients seeing generalists only (6%). This distribution differed from that of another system in the same metropolitan area where 57% of HIV-infected patients were followed in ID, 17% were comanaged by an ID and generalist provider, and 26% saw a generalist with HIV training[22]. In 1996, 46% of PLWHA were cared for by ID providers, 45% by self-identified expert generalists, and 9% by non-expert generalists[52]. In 2015, HealthHIV’s State of HIV in Primary Care in the US showed HIV providers to identify their specialty as HIV/AIDS (30%), family medicine (20%), infectious disease (17%), internal medicine (15%)[21].

Our study’s HIV care models differed on the basis of patient population served, varying by sex, race, and primary language. Compared with the other two groups, the ID group had higher proportions of white, English speaking, and male patients, and lower rates of patients meeting IOM retention criteria. The generalist group had higher proportions of Hispanic and non-English speaking patients with lower median Charlson comorbidity scores than ID or ID plus generalist groups. The ID plus generalist group had higher proportions of black patients, had higher median clinic visits, and higher median Charlson comorbidity scores than ID or generalist groups. These distinct populations may reflect patient preference and self-selection: clinics and/or providers may be more or less accessible or preferable to patients based on factors such as geography, public transportation, or language skills of providers. Patients cared for through the different models, however, did not differ on the basis of most clinical HIV markers, including CD4 cell count, nadir CD4 cell count, or ART use; these finding are consistent with prior studies[22]. Patients in different HIV care models also varied by comorbidity burden and number of visits: the ID plus generalist group had significantly more co-morbidities and more clinic visits, although were not older. This pattern could indicate that as patients with HIV develop more co-morbidities, they are more likely to participate, either through self-selection or provider choice, in an HIV care model with both generalists and ID providers.

In an exploratory analysis examining DM by individual screening tests, we found different practice patterns comparing ID and ID plus generalist groups: ID plus generalist were more likely to screen for diabetes by A1C and the ID group was more likely to screen by fasting glucose. These findings might reflect an increased awareness among ID providers of literature suggesting that A1C underestimates average fasting glucose in the setting of HIV infection[53–55] which may influence choice of screening methods for diabetes.

While HIV-related screening and outcome measures were not the focus on this analysis, we found that rates of viral suppression differed by HIV care model in preliminary analyses, with the generalist group having significantly lower rates of viral suppression compared to the ID and ID plus generalist groups. Prior studies have shown that generalists provide equivalent care with regards to HIV outcomes[49, 50]; however, our sample did not classify providers by levels of experience, previously shown to impact HIV outcomes[26, 44–46]. Our sample size for generalists is less than 6% of the overall sample, however, and reflects clinical practice patterns of many fewer providers compared with the ID and ID plus generalist groups. Ongoing further studies in this cohort will examine this finding in greater depth and assess a wider distribution of HIV quality of care metrics.

Limitations of our study include the fixed population size that limits our power to detect differences between HIV care models. This could be addressed with a multi-year study analysis in our longitudinal database. The ability to generalize to other populations is also limited given the variation in HIV care models geographically and by population density in rural vs. urban areas. However, we used a large system wide cohort with rich data that examines a question that existing national databases cannot currently address. Our analysis may also be subject to misclassification in exposure groups; however, we conducted rigorous validation studies to optimize accuracy of primary exposure classification, correctly classifying 94% in our validation sample. To account for possible measurement bias in outcomes that were not captured via EMR, we utilized previously validated algorithms for CRC, breast cancer, and cervical cancer to ensure the validity of outcomes and excluded patients with outside providers. If measurement bias is present, we would not expect it to be different among exposure groups.

In conclusion, we demonstrate in a large urban cohort of PLWHA that there are no significant differences in metabolic/CVD or cancer screening rates by HIV care model, after adjusting for sociodemographic and clinical factors and accounting for clustering by physician provider. Overall, our findings suggest that patients are receiving similar preventive health care for NCDs whether cared for by ID, generalist, or ID and generalist providers jointly. Identifying the optimal HIV care model is an issue with significant public health relevance as PLWHA age and confront an increasing burden of non-HIV-related chronic disease complications.

## Supporting Information

S1 Appendix Exposure Classification and Model Building (Methods A). Targeted Chart Review Methods **(Table A)**. Cancer Screening Outcome Classification Data **(Table B)**. Unadjusted and Adjusted odds ratios for Outcomes **(Table C)**.
(DOCX)
