# Supplementary material for: Non-Communicable Disease Preventive Screening by HIV Care Model
Source: PLoS One. 2017 Jan 6;12(1):e0169246. doi: 10.1371/journal.pone.0169246 (PMC5218477; doi:10.1371/journal.pone.0169246)
Supplement: S1 Appendix — Targeted Chart Review Methods (Table A). Cancer Screening Outcome Classification Data (Table B). Unadjusted and Adjusted odds ratios for Outcomes (Table C). (DOCX) [file pone.0169246.s001.docx]

**Supporting Appendix**

**Supporting Methods**

**Exposure Classification**

The primary exposure of interest was type of HIV care model based on clinic visits to the corresponding type of provider(s) between 1/1/2011 and 12/31/2012: (1) infectious disease provider only (ID), (2) generalist provider only (generalist), or (3) infectious diseases and generalist providers (ID plus generalist). Provider specialty was classified as ID or generalist by electronic medical record (EMR) categorization and all provider specialties were validated by physician review using institutional biographies and fellow rosters.

As EMR-listed PCP were known to be unreliable or outdated, we developed and validated an HIV care model classification algorithm. Initially, HIV care model classification was determined by number of visits to ID and/or generalist providers over a two year period, with ID model defined as >0 visits to ID provider only, generalist model defined as >0 visits to generalist provider only, and ID plus generalist model defined as both >0 visits to an ID and >0 visits to a generalist provider. This yielded 1345 patients in ID model, 162 patients in generalist model, and 534 in ID plus generalist model in a total cohort of 2041 (Figure 1).

To validate this method, we randomly selected and reviewed 75 charts from the 2041 cohort with correct classification in 68% of patients, determined by physician review of 2011-2012 clinic visits and search for PCP listed in notes using natural language searching software Queriable Patient Inference Dossier (QPID) technology. Therefore, HIV care model classifications underwent targeted physician chart review and reclassification, with specific review of 4 subgroups, as seen in S1 Table. **S1 Table: Targeted Chart Review Methods**

| Group Reviewed | Goal of Review | Review Method |
| --- | --- | --- |
| Generalists | Identify and remove generalist patients receiving only urgent care | Review 2011-2012 generalist clinic notes |
| ID plus generalists with 1 generalist visit in 2012 | Identify and reclassify ID patients incorrectly classified due to urgent care visit to generalist | Review 2011-2012 generalist clinic notes |
| EMR-listed PCP not PHS provider, incongruent with classification, or missing | Either 1) identify and remove patients who do not meet only PHS providers inclusion criteria or 2) reclassify incorrect exposure group | Review 2011-2012 clinic notes and use QPID search for EMR-listed or non-PHS PCP |
| Patients missing structured EMR vital signs | Identify and remove patients without clinical encounter in 2012 (i.e. patients with only medication reconciliation visits) | Review 2012 clinic notes |

Review of these groups identified patients who did not meet inclusion criteria of 1 visit to HIV or generalist provider in 2012 or who met exclusion criteria with HIV provider outside PHS in 2011-2012. After re-categorization, the final patient distribution was 875 ID model, 90 generalist model, and 600 ID plus /generalist model.

We performed a chart review of 100 randomly selected patients from this final cohort to determine the accuracy of HIV care model categorization and ascertained correct exposure classification in 94% of patients.

**Model Building**

For the multivariate analysis we assumed a compound correlation structure. Below we list the coefficients and confidence intervals for all covariates incorporated into the model. The model that we present in the final analysis is a prespecified model with covariates chosen based on clinical knowledge. We also explored additional generalized estimating equation (GEE) modeling approaches including purposeful selection and random effects logistic regression models that had similar final results.

**S2 Table: Cancer Screening Outcome Data**

|  | Colorectal Cancer | Cervical Cancer | Breast Cancer |
| --- | --- | --- | --- |
| Patient Sex | Male and female | Female | Female |
| Patient Age | 50-75 | 18-75 | 50-75 |
| Exclusion Criteria | Colectomy | Hysterectomy | Double Mastectomy |
| Exclusion Criteria sources | Problem lists, CPT and ICD-9 codes | Problem lists, CPT and ICD-9 codes | Problem lists, CPT and ICD-9 codes |
| Exclusion ICD-9 and CPT codes | 45.8, 45.81-3, 441.50-3,  441.55-8,  442.10-2 | 68.4-9, 519.25, 585.48, 585.50, 585.52-54, 563.08, 595.25, 581.50, 581.52, 581.80, 582.00, 582.10, 582.40, 582.60, 582.62-3, 582.67, 582.70, 582.75, 582.80, 582.85, 582.90-4, 585.70-3, 589.51, 589.53-4, 589.56, 591.35 | 85.41-8, 191.80, 192.00, 192.20, 192.40,  193.03-7 |
| Screening Guidelines | IDSA Primary Care | IDSA Primary Care | USPSTF |
| Screening test  (screening interval) | Colonoscopy (10 years), Flexible Sigmoidoscopy (5 years), Virtual Colonoscopy (5 years), Double Barium (5 years) | Papanicolauou Smear (annually) | Mammogram (biannually) |
| Screening test sources | Health Maintenance EMR coded data, CPT and ICD-9 codes | Health Maintenance EMR coded data, CPT and ICD-9 codes | Radiology data, Health Maintenance EMR coded data, CPT and ICD-9 codes |
| Screening ICD-9 and CPT codes | 45.22-5, 45.42-3,  443.88-94, 443.97, 453.30-35, 453.37-42, 453.45, 453.55,  453.78-88, 453.91-2, 742.70, 742.80, 0066T, 0067T, G0104-6,  G0120-22 | 91.46, 876.20-2,  881.41-3, 881.47, 881.50, 881.55,  881.60-2, 881.64-5, 881.72-5, G0123-4, G0141, G0143-5, P3000-1, Q0091 | 87.36-7, 760.85,  760.90-2, 770.51-2, 770.55-59, G0202-7 |

**S3 Table: Unadjusted and Adjusted Odds Ratios for Outcomes**

|  | OR (95%CI) Unadjusted | OR (95%CI) Adjusted* |
| --- | --- | --- |
|  |  |  |
|  | Obesity | |
| ID n=875 | Reference | Reference |
| Generalist n=90 | 0.96 (0.43-2.16) | 0.67 (0.26-1.72) |
| ID&Generalist n=600 | 1.84 (1.29-2.61) | 1.27 (0.90-2.00) |
|  | Hyperlipidemia | |
| ID n=875 | Reference | Reference |
| Generalist n=90 | 1.32 (0.72-2.43) | 1.41 (0.81-2.46) |
| ID&Generalist n=600 | 1.22 (0.93-1.60) | 0.95 (0.71-1.28) |
|  | Diabetes | |
| ID n=733 | Reference | Reference |
| Generalist n=74 | 1.12 (0.50-2.52) | 0.98 (0.44-2.19) |
| ID&Generalist n=448 | 1.01 (0.78-1.31) | 0.86 (0.67-1.11) |
|  | A1C | |
| ID n=733 | Reference | Reference |
| Generalist n=74 | 1.59 (0.69-3.62) | 1.42 (0.57-3.58) |
| ID&Generalist n=448 | 1.38 (1.01-1.87) | 1.24 (0.87-1.75) |
|  | Fasting Glucose | |
| ID n=733 | Reference | Reference |
| Generalist n=74 | 0.75 (0.30-1.89) | 0.70 (0.27-1.82) |
| ID&Generalist n=448 | 0.79 (0.60-1.03) | 0.69 (0.53-0.90) |
|  | Colorectal Cancer | |
| ID n=451 | Reference | Reference |
| Generalist n=39 | 0.94 (0.33-2.65) | 0.99 (0.23-4.23) |
| ID&Generalist n=324 | 1.22 (0.91-1.62) | 0.93 (0.67-1.30) |
|  | Cervical Cancer | |
| ID n=191 | Reference | Reference |
| Generalist n=26 | 1.77 (0.77-4.08) | 3.08 (0.80-11.86) |
| ID&Generalist n=196 | 1.06 (0.63-1.78) | 1.06 (0.61-1.85) |
|  | Breast Cancer | |
| ID n=76 | Reference | Reference |
| Generalist n=14 | 1.97 (0.66-5.85) | 0.60 (0.16-2.20) |
| ID&Generalist n=102 | 1.71 (0.93-3.16) | 1.27 (0.62-2.59) |

*Adjusted for age, sex, race, language, number of 2012 clinic visits, median household income, viral load, most recent CD4 cell count, and weighted Charlson score.
